# Growth Parameters of Various Green Microalgae Species in Effluent from Biogas Reactors: The Importance of Effluent Concentration

**DOI:** 10.3390/plants11243583

**Published:** 2022-12-19

**Authors:** Elvira E. Ziganshina, Svetlana S. Bulynina, Ksenia A. Yureva, Ayrat M. Ziganshin

**Affiliations:** Department of Microbiology, Institute of Fundamental Medicine and Biology, Kazan (Volga Region) Federal University, 420008 Kazan, Republic of Tatarstan, Russia

**Keywords:** green microalgae, photobioreactor, anaerobic digester effluent, nutrient recycling, algal biomass, proteins

## Abstract

The use of liquid waste as a feedstock for cultivation of microalgae can reduce water and nutrient costs and can also be used to treat wastewater with simultaneous production of biomass and valuable products. This study applied strategies to treat diluted anaerobic digester effluent (ADE) as a residue of biogas reactors with moderate (87 ± 0.6 mg L^−1^; 10% ADE) and elevated NH_4_^+^-N levels (175 ± 1.1 mg L^−1^; 20% ADE). The effect of ADE dilution on the acclimatization of various microalgae was studied based on the analysis of the growth and productivity of the tested green algae. Two species of the genus *Chlorella* showed robust growth in the 10–20% ADE (with a maximum total weight of 3.26 ± 0.18 g L^−1^ for *C. vulgaris* and 2.81 ± 0.10 g L^−1^ for *C. sorokiniana*). The use of 10% ADE made it possible to cultivate the strains of the family *Scenedesmaceae* more effectively than the use of 20% ADE. The growth of *Neochloris* sp. in ADE was the lowest compared to other microalgal strains. The results of this study demonstrated the feasibility of introducing individual green microalgae into the processes of nutrient recovery from ADE to obtain biomass with a high protein content.

## 1. Introduction

Green microalgae are recognized as a promising feedstock for obtaining valuable products and can also serve as resources with a high potential for bioassimilation and adsorption of various compounds [1,2]. Cultivation of microalgae requires light, carbon dioxide, nutrients (nitrogen, phosphorus, and sulfur), and various trace elements. The cost of nutrients for the cultivation of microalgae is one of the factors contributing to the high cost of algae biomass and products derived from it. Today, many scientific groups consider the cultivation of microalgae in wastewater as an economically and environmentally sustainable approach to algal biotechnology. The application of this approach will reduce the risks associated with the high cost of nutrients and large volumes of water required for the growth of algae [3,4,5,6,7].

Compared to existing wastewater treatment methods, biological purification of water using microalgae can have many economic and environmental benefits [2]. However, the researchers note that not all strains can provide a high potential for survival in non-ideal environments, as well as the fact that wastewater is not always a feasible alternative for the cultivation of algae. The abundance of certain substances, including toxic ones, and the lack of some macro- and micronutrients, as well as the dark color of wastewater, can be a problem for algae growth [6,8,9]. In addition, research on the bioremediation of waste by microalgae usually focuses on small laboratory-scale tests of a narrow range of model species and rarely includes real wastewater streams, which certainly underlines the relevance of these studies.

The anaerobic digestion process is widely used in the treatment of organic wastes with the simultaneous production of biogas [10,11,12]. Such biological treatment of waste materials can lead to a reduction in greenhouse gas emissions [13] and provide a residual digestate as a by-product, which must be disposed of to prevent harm to the environment. The presence of residual concentrations of the main biogenic elements (carbon, nitrogen, and phosphorus) and various micronutrients in the anaerobic digester effluent [14] can potentially serve as a medium for the cultivation of microalgae [15,16,17]. Among the works in this area of research, we note the works in which digestates after anaerobic conversion of kitchen waste [6,9], municipal wastewater [18], and agricultural waste [6,18,19,20,21,22] were then treated by various microalgae.

At the industrial level, preference is given to more resistant and fast-growing algal species, for example, those belonging to the genera *Chlorella*, *Scenedesmus*, and *Nannochloropsis*. These microalgae have usually been proposed as candidates for waste processing, and their biomass can serve as a source of energy and different useful products, such as biofertilizers and animal feed [6,17]. Researchers in this field note that for the successful cultivation of microalgae in anaerobic digester effluent, pre-treatment of the digestate is necessary, including sedimentation and microfiltration [6,16]. Fernandez et al. [6] used processed digestates (after processing of kitchen and agricultural wastes) as a nutrient medium for the cultivation of *Chlorella vulgaris* and *Scenedesmus obliquus*. In another work [18], for the cultivation of *C. vulgaris* and *Scenedesmus* sp., the authors tested various digestates from anaerobic reactors that treated manure/silage, sludge, and municipal wastewater. Another recent study also confirmed the great potential of cultivating green microalgae and cyanobacteria on the digestate from an agricultural biogas plant that was operated with maize silage and distillery stillage, as well as the importance of waste pretreatment to produce valuable biomass [20]. Therefore, researchers in this area are convincingly considering certain strains of microalgae that can efficiently grow and treat wastewater, agricultural waste, sludge, and residual digestate. However, such research needs to be continued using new strains of microalgae and new substrates to reduce the negative impact of waste on the environment.

Thus, to reduce economic costs and the impact of microalgae cultivation on the environment, it is essential to use alternative low-cost substrates such as effluents from biogas reactors, avoiding the need for additions of chemical compounds and large volumes of clean water. The aim of the study was to determine the ability of various new strains of microalgae to utilize nutrients in digestate from biogas reactors and to determine the effect of using different concentrations of digestate on the growth and productivity of the tested algae. Since sterilization of wastewater is not a cost-effective option for mass culture, unsterilized digestate was used in this work. Newly isolated strains of microalgae *Chlorophyta* (*Chlorella sorokiniana*, *Chlorella vulgaris*, *Tetradesmus obliquus*, *Scenedesmaceae* sp. and *Neochloris* sp.) were tested as potential agents for biomass accumulation and water purification. Furthermore, the protein level was analyzed to determine the potential of microalgae grown on digestate as a valuable product for further application.

## 2. Results and Discussion

### 2.1. Tested Microalgal Species

**Figure 1:**
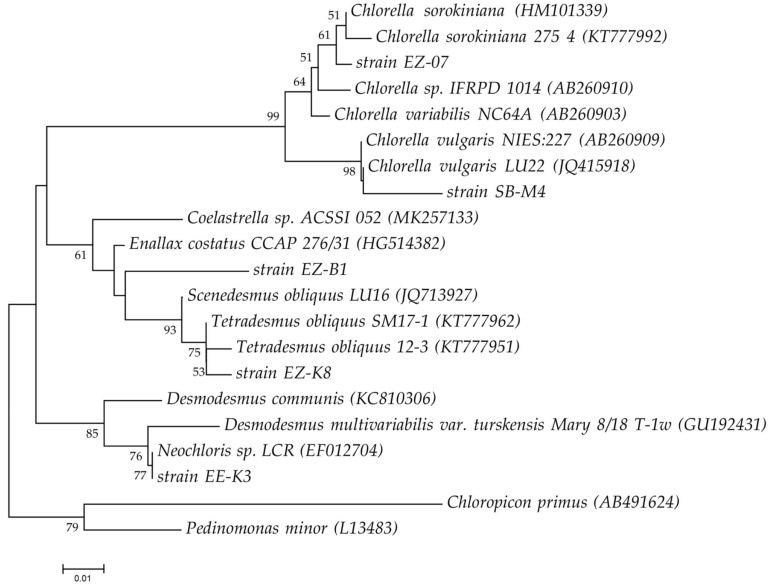
The evolutionary history was inferred using the neighbor-joining method (based on *rbcL* gene sequences). The percentage of replicate trees in which the associated taxa clustered together in the bootstrap test (1000 replicates) are shown next to the branches. The tree is drawn to scale, with branch lengths in the same units as those of the evolutionary distances used to infer the phylogenetic tree. The evolutionary distances were computed using the Kimura 2-parameter method and are in the units of the number of base substitutions per site. The analysis involved 21 nucleotide sequences. Evolutionary analyses were conducted in MEGA7 [23]. *Chloropicon primus* and *Pedinomonas minor* were used as outgroup references.

This study investigated the effect of two different concentrations of anaerobic digester effluent (ADE) on the algal growth, biomass accumulation, as well as metabolite production by newly isolated microalgae belonging to the families *Chlorellaceae*, *Scenedesmaceae*, and *Neochloridaceae*. All five strains of green microalgae were isolated from various freshwater reservoirs in the city of Kazan (the Republic of Tatarstan, Russia). The phylogenetic analysis of isolated algal species and other algae used for comparison in this study is demonstrated in Figure 1.

All microalgal species were compared based on the sequences of the ribulose bisphosphate carboxylase large subunit (*rbcL*) gene. The *rbcL* gene of the strain EZ-07 shared 97–100% BLAST identity with the *rbcL* gene sequences of various *Chlorella sorokiniana* strains, whereas the *rbcL* gene of the strain SB-M4 shared 99% BLAST similarity with the *rbcL* sequences of different *Chlorella vulgaris* strains. The *rbcL* gene sequence of the strain EZ-K8 had 100% BLAST identity to *rbcL* of *Tetradesmus obliquus*. The strain EZ-B1 shared 95% BLAST identity with the *rbcL* of *Coelastrella saipanensis* and *Enallax costatus*, while the strain EE-K3 shared 99% and 98% BLAST identities with the *rbcL* sequences of *Neochloris* sp. and *Desmodesmus multivariabilis var. turskensis*, respectively. Based on the morphological and sequence data, the tested strains of microalgae were assigned to *Chlorella sorokiniana* EZ-07 (*Chlorellaceae*), *Chlorella vulgaris* SB-M4 (*Chlorellaceae*), *Tetradesmus obliquus* EZ-K8 (*Scenedesmaceae*), *Scenedesmaceae* sp. EZ-B1, and *Neochloris* sp. EE-K3 (*Neochloridaceae*). The *rbcL* gene sequence of the strain EZ-B1 had low matches in the National Center for Biotechnology Information (NCBI) database, and therefore this strain may represent a new algal species.

### 2.2. Growth Parameters of Microalgae in ADE

Anaerobic digester effluent was obtained from laboratory biogas reactors in which distillers’ grains with solubles and cow manure were processed under mesophilic conditions. The undiluted digestate was dark, resulting in reduced light availability, and was too concentrated for efficient algal growth. As a result, the ADE used in this study was centrifuged to remove solid particles and increase light penetration for efficient microalgae growth. Table 1 describes the main characteristics of the initial digestate. As a culture medium, two concentrations of anaerobic digestate were chosen, referred to as 10% ADE and 20% ADE. The processed ADE was diluted to reach an ammonium nitrogen (NH_4_^+^-N) concentration of 87 ± 0.6 mg L^−1^ (10% effluent loading) and 175 ± 1.1 mg L^−1^ (20% effluent loading). In addition, phosphate, sulfate, and potassium levels were adjusted to the concentrations of the standard Bold’s basal medium [24].

**Figure 2:**
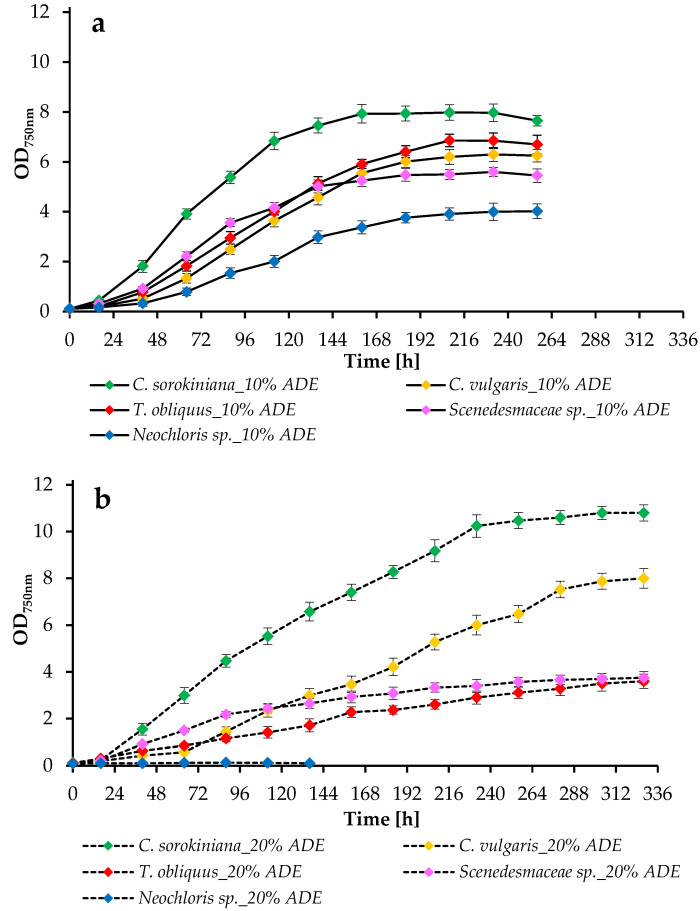
Growth characteristics of algal cultures: (**a**) growth curves of microalgae cultured in 10% ADE; (**b**) growth curves of microalgae cultured in 20% ADE.

**Figure 3:**
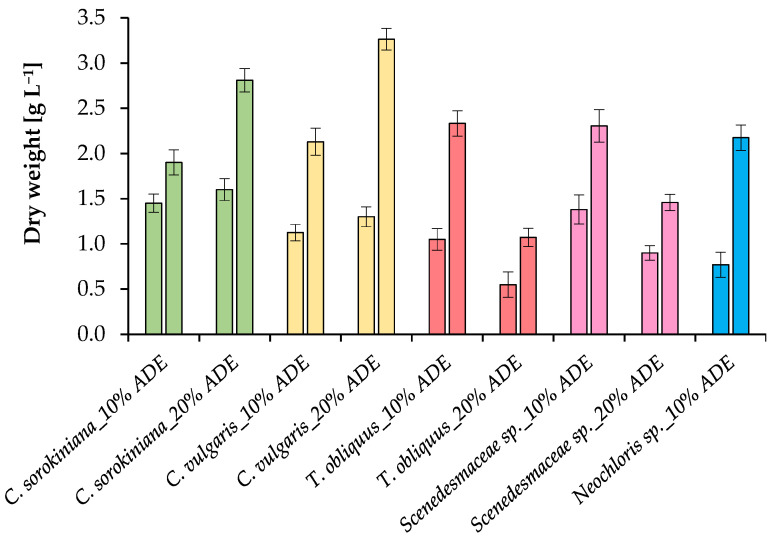
Growth characteristics of algal cultures: dry weight of microalgae cultured in 10% ADE (measured on 88 h and 256 h) and 20% ADE (measured on 136 h and 328 h).

**Figure 4:**
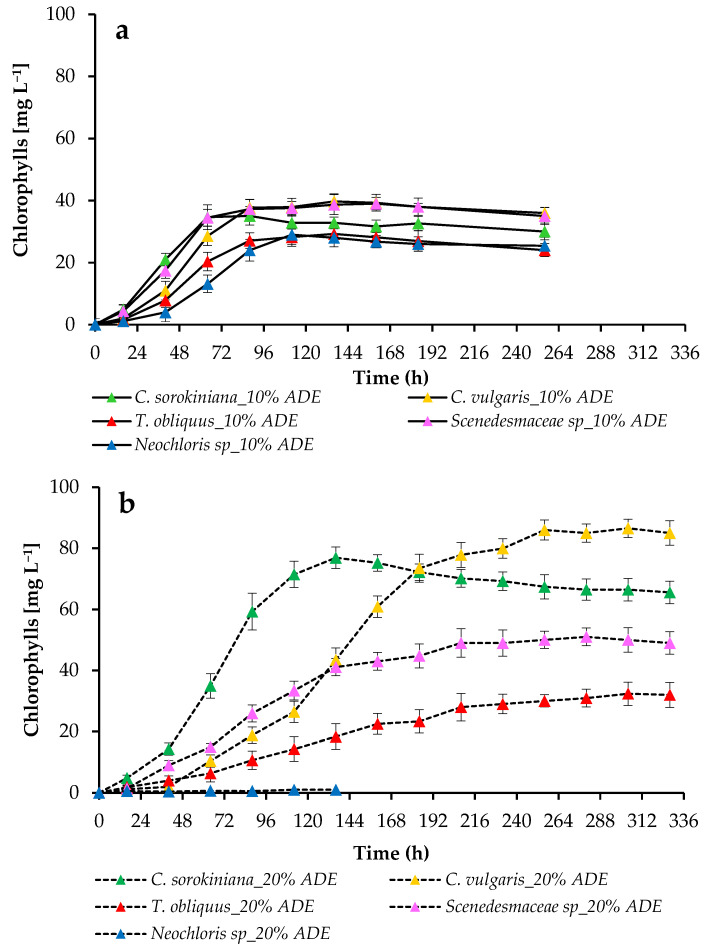
Chlorophylls concentration for cultures grown in 10% ADE (**a**) and 20% ADE (**b**).

The growth pattern of the tested algal cultures under different conditions was controlled by measuring the optical density at 750 nm (OD_750nm_) (Figure 2), the dry weight of microalgae (Figure 3), and the absorption of pigments (Figure 4). The productivity values and growth kinetics showed good adaptability of individual cultures to higher levels of ADE and ammonium nitrogen as well as reduced light availability (Table 2).

The obtained data indicate that although all investigated species showed nice growth when cultured in 10% ADE, only two of the tested species were able to maintain sustained growth in harsh environmental conditions with unsterilized 20% ADE. For example, *Neochloris* sp. EE-K3 cultures were destroyed in 20% ADE after a few days of cultivation. However, two species of the genus *Chlorella* demonstrated robust growth in the same environment, including resistance and high growth rates.

The growth curves of *C. sorokiniana* EZ-07 illustrated in Figure 2 indicate faster growth when cultured in 10% ADE and 20% ADE compared to the other tested strains. Cells of the strain EZ-07 in the experiments with 10% effluent loading reached the stationary phase by 160 h of cultivation, while cells cultivated in 20% ADE, although they had a slight decrease in initial growth, were able to overtake in optical density measurements and total weight (2.81 ± 0.10 g L^−1^ at 20% ADE loading versus 1.90 ± 0.14 g L^−1^ at 10% ADE loading) (Figure 3). This was probably due to the increased content of ammonium and other components in the 20% ADE-based medium.

In the case of *C. vulgaris* SB-M4, the growth curves show faster culture growth when cultured in a medium based on 10% ADE. Whereas when grown in 20% ADE, the culture SB-M4 takes longer to acclimatize to the higher effluent content in the growth medium (the maximum OD_750 nm_ values were noted at 232 h and 328 h of cultivation, respectively) (Figure 2). The SB-M4 culture achieved final dry weights of 2.13 ± 0.13 g L^−1^ and 3.26 ± 0.18 g L^−1^ at 10% ADE and 20% ADE loadings, respectively (Figure 3).

The growth characteristics of two representatives of the family *Scenedesmaceae* (*T. obliquus* EZ-K8 and *Scenedesmaceae* sp. EZ-B1) in the medium with 20% effluent loading were lower compared to the experiments performed with 10% ADE. Thus, the growth curves of these microalgae when grown in 20% liquid digestate are characterized by a long exponential phase, which may indicate the inhibition of microalgae by the ADE composition (Figure 2). The strain EZ-K8 achieved final dry weights of 2.33 ± 0.15 g L^−1^ and 1.07 ± 0.09 g L^−1^ at 10% ADE and 20% ADE loadings, respectively. The strain EZ-B1 reached final dry weights of 2.31 ± 0.12 g L^−1^ and 1.46 ± 0.14 g L^−1^ at 10% ADE and 20% ADE loadings, respectively (Figure 3).

In the case of *Neochloris* sp. EE-K3, the growth values in 10% ADE were the lowest compared to other strains. The strain EE-K3 did not grow when treated with 20% ADE, and the cultivation was aborted after a few days (Figure 2). The strain EE-K3 reached a final dry weight of 2.17 ± 0.14 g L^−1^ in experiments with 10% ADE (Figure 3).

It should be noted that the cells of the tested cultures have different morphology and size, which may affect the values of optical density and dry weight. On the one hand, smaller algal cells show a higher optical density compared to larger cell forms due to higher scattering. On the other hand, dry mass measurement is not suitable for daily monitoring. To overcome these limitations, different methods must be combined to improve information content and reliability [25]. Furthermore, an additional contribution to these data was made by some bacteria that grew in the presence of microalgae. Therefore, in addition to OD_750 nm_ and dry weight measurements, we additionally estimated the number of cells using a counting chamber (Table 2).

The total chlorophyll content of the cultures was measured during the experimental period to investigate the photosynthetic potential of each microalgal culture. When analyzing the content of chlorophylls *a* and *b* of the cultures grown in 10% ADE-based medium, we observed that during the first hours of cultivation, the content of chlorophylls in *C. sorokiniana* EZ-07 and *Scenedesmaceae* sp. EZ-B1 were higher than the content of chlorophylls in the other strains (Figure 4a). After 64–136 h (depending on the culture), a partial decrease in the accumulation of chlorophylls *a* and *b* in microalgal cells was noted. Finally, cells of the strains SB-M4 and EZ-B1 had the highest chlorophyll content, while cells of the strains EZ-K8 and EE-K3 had the lowest chlorophyll content when cultured at a 10% effluent loading regimen. 

In experiments with 20% ADE, *C. sorokiniana* EZ-07 and *C. vulgaris* SB-M4 demonstrated the highest content of chlorophylls *a* and *b*, although *C. sorokiniana* EZ-07 accumulated them faster. The lowest content of chlorophylls *a* and *b* was also observed in *T. obliquus* EZ-K8 cultured in 20% ADE (Figure 4b). This may be due to both the features of the photosynthetic apparatus and the culture’s need for nutrients. Considering that chlorophyll is an available intracellular pool of nitrogen, which algae can use for cell growth after nitrogen depletion in the culture medium [26], a substantial decrease in chlorophyll content was noted in *C. sorokiniana* EZ-07. This highlights the lack of nitrogen in culture medium for this strain.

When analyzing the content of carotenoids in microalgal cells during the growth in 20% ADE, the highest accumulation of carotenoids was observed in *C. vulgaris* and *C. sorokiniana* species (Table 2). Carotenoids are synthesized by microalgae as primary metabolites to protect photosystems from photodamage and to broaden the light harvesting spectrum and as secondary metabolites under stress conditions. Thus, data on carotenoids can serve as indicators of cell function and help select promising sources of carotenoid-rich extracts [27].

This study confirmed the need for digestate pretreatment for the effective growth of *C. sorokiniana*, *C. vulgaris*, *T. obliquus*, *Scenedesmaceae* sp., and *Neochloris* sp. and the need to screen microalgae for digestate processing. All tested strains were satisfied with the growth medium prepared by centrifugation of digestate after anaerobic digestion of distillers’ grains with solubles/cow manure and a subsequent 1:10 dilution, while the more concentrated effluent only stimulated the growth of *Chlorella* species. There was a significant difference between the final mean biomass yield of microalgae cultivated in 10% and 20% ADE. The biomass productivity of *Chlorella* strains in 10% and 20% ADE was found to be similar (in the range of 0.26–0.30 g L^−1^ day^−1^), while the strains of the *Scenedesmaceae* achieved a maximum biomass productivity of 0.15 ± 0.01 g L^−1^ day^−1^ when 20% effluent was used. Despite the lower biomass production in 20% ADE, the results demonstrated that both *T. obliquus* and *Scenedesmaceae* sp. can grow in the liquid, unsterilized digestate, while a higher digestate concentration completely inhibits *Neochloris* sp. growth. This may be attributed to the stronger color of the medium and the physiological characteristics of the strains. The results showed that 20% ADE yielded the best dry weight for both *Chlorella* species—*C. sorokiniana* (2.81 ± 0.10 g L^−1^) and *C. vulgaris* (3.26 ± 0.18 g L^−1^).

Comparing our results with the works of other authors, we can note the following works. For example, in another work [6], the authors reached a maximum concentration of *C. vulgaris* biomass of 0.49 g L^−1^ and *Scenedesmus* biomass of 0.23 g L^−1^ in pig manure-based digestate when cultivated in flasks, while Kisielewska et al. [20] obtained a maximum final concentration of *C. vulgaris* biomass of 2.49 g L^−1^ in media based on centrifuged liquid agricultural digestate in tubular photobioreactors. Chen et al. [28] demonstrated that the highest biomass concentration (5.45 g L^−1^) of *C. sorokiniana* can be obtained during cultivation in 50%-strength filtered swine wastewater in photobioreactors (glass-made vessels). Bohutskyi et al. [29] noticed that most of the tested species except several *Chlorella* and *Scenedesmus* species could not grow efficiently in wastewater and the liquid fraction of the anaerobic digestion effluent in cylindrical bioreactors. However, comparison of achieved biomass yields with data from other works should be accurate due to various parameters (in particular, light intensity, CO_2_ supply, temperature, experiment duration, and type of bioreactor), as well as the origin and characteristics of the digestate, which directly affect the growth of algae. Our results are also consistent with the results of other studies, which stated that the species of the genus *Chlorella* are among the most effective when grown in various wastewater systems [18,30].

Literature data also show that dilution of liquid digestate is an important factor in producing microalgal biomass [6,20,31]. The use of ethyl alcohol production residues and dairy farm wastes (cow manure) in this study resulted in a digestate with a moderate dry matter content (Table 1). However, centrifugation and dilution of digestate reduced the nutrient concentration in the culture medium, which may explain the lower growth rate of the individual strains in the ADE-based media. Therefore, it is important to optimize the balance of nutrients in such media. The composition of diluted digestate was further adjusted to support phosphate, sulfate, and potassium levels. However, the reduced growth of individual microalgae may be due to other micronutrient deficiencies. Differences in nutrient concentrations in digestate-based media were shown by Kisielewska et al. [20], where the authors tested different methods of digestate processing. The authors confirmed that the culture media prepared by centrifugation of digestate from an agricultural biogas plant for further cultivation of algal strains, including *C. vulgaris*, contained much higher concentrations of total phosphorus and organic compounds compared to the media prepared by distillation. The results of other studies confirm that the use of high concentrations of digestate increases the turbidity of the growth medium, limiting the availability of light for photosynthesis and affecting the production of biomass [6,17]. It is also important to consider that the anaerobic digestate analyzed in this work was not sterilized before use, as the focus of this research was on the possibility of using a non-sterile effluent to support the cultivation of various microalgae within *Chlorophyta*. Although the presence of competing heterotrophic bacteria may reduce the productivity of algae biomass in photobioreactors, the use of additional digestate preparation procedures will increase the cost of cultivation on an industrial scale.

### 2.3. Ammonium Nitrogen Uptake by Strains and pH Change during Cultivation at Different Effluent Loads

**Figure 5:**
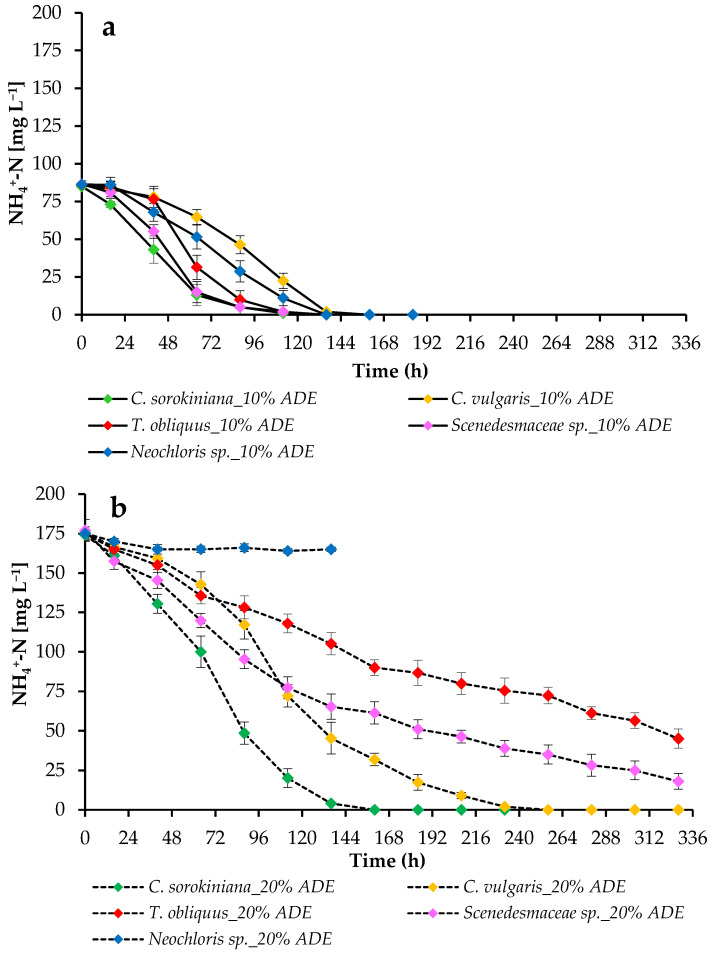
Ammonium nitrogen concentrations in the medium during the growth of microalgae in 10% ADE (**a**) and 20% ADE (**b**).

Since microalgal biomass production is driven by nutrient uptake, the degree of ammonium nitrogen removal from the ADE-based medium was estimated (Figure 5).

The highest NH_4_^+^-N utilization in 10% ADE was achieved by cells of *C. sorokiniana* EZ-07 and *Scenedesmaceae* sp. EZ-B1. Three other strains also efficiently consumed NH_4_^+^-N, but with a delay. For all strains, 100% nitrogen removal in 10% ADE was noted at 136 h of cultivation (Figure 5a). An increase in the concentration of ADE led to partial inhibition of the growth of *T. obliquus* EZ-K8 and *Scenedesmaceae* sp. EZ-B1 and complete growth inhibition of *Neochloris* sp. EE-K3. These strains could not use all the available NH_4_^+^-N. Only *C. sorokiniana* EZ-07 and *C. vulgaris* SB-M4 were able to maintain sustained growth in the harsh environmental conditions with unsterilized 20% ADE and consume all the available NH_4_^+^-N (Figure 5b). 

**Figure 6:**
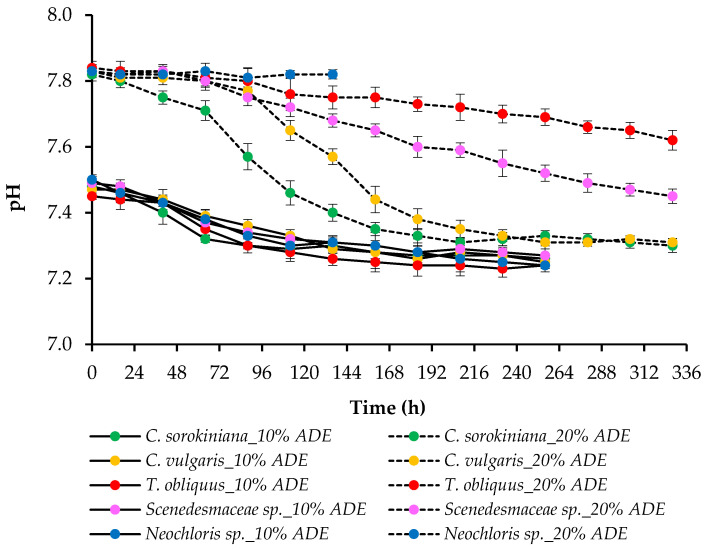
pH changes during the growth of microalgae in 10% ADE and 20% ADE.

Since the uptake of NH_4_^+^ by algae decreases the pH value due to the equimolar release of H^+^ [32], the pH values changed from the initial 7.45–7.50 to the final 7.25–7.30 in 10% ADE and from the initial 7.80–7.85 to the final 7.30–7.60 in 20% ADE. A higher rate of NH_4_^+^-N removal led to a stronger drop in the pH of the medium (Figure 6). Even though we cultivated algae in the unsterilized effluent, the main role in the utilization of ammonium belonged to algae since most of the microorganisms were removed during the preparation of the effluent and their remaining level was negligible.

The current study evaluated the removal of NH_4_^+^-N from ADE-based culture media by algae. After 136 h of cultivation, all strains achieved 100% nitrogen removal in 10% ADE. The highest NH_4_^+^-N removal rate in 20% ADE was achieved during cultivation of *C. sorokiniana* EZ-07, while the efficiency of other strains decreased as the concentration of ADE increased. In harsh environmental conditions, only tested *Chlorella* strains were able to sustain growth and consume all available ammonium.

In another work [31], various species of microalgae, including *Neochloris oleoabundans*, *C. vulgaris*, and *S. obliquus*, were cultivated in an agro-zootechnical digestate under comparable conditions. *C. vulgaris* and *N. oleoabundans* showed the ability to remove up to ~160 mg L^−1^ of NH_4_^+^-N from diluted digestate samples. Zheng et al. [33] cultivated *C. vulgaris* in piggery wastewater with different NH_4_^+^ concentrations and determined the optimal NH_4_^+^ concentration for microalgal growth at 110 mg L^−1^. When comparing the growth rates of other *Chlorella* species and *S. obliquus* in autoclaved anaerobically digested swine wastewater at various concentrations [34], it was found that the most efficient accumulation of biomass occurred at the lowest tested concentration of digestate. Despite this, the cultures were able to grow in more aggressive environments, experiencing significant growth inhibition and NH_4_^+^-N utilization ability in 80–100% ADE.

The degree of resistance of microalgae to NH_4_^+^/NH_3_ is different in various algal classes, and *Chlorophyceae* are much more resistant to elevated NH_4_^+^ concentrations than other unicellular algae [32]. However, in this work, even within the same class, different resistances of individual microalgal species to an increased level of ammonium nitrogen in the digestate were revealed. Apparently, the increased growth of representatives of the genus *Chlorella* at a 20% ADE loading regimen can be also associated with their tolerance to high concentrations of NH_4_^+^. Considering that in our systems the temperature was maintained at 28 °C and the pH did not exceed 8.0, the toxicity for distinct strains might be related to ammonium ions rather than to free ammonia.

### 2.4. Comparison of Protein Contents in Algal Cells

**Figure 7:**
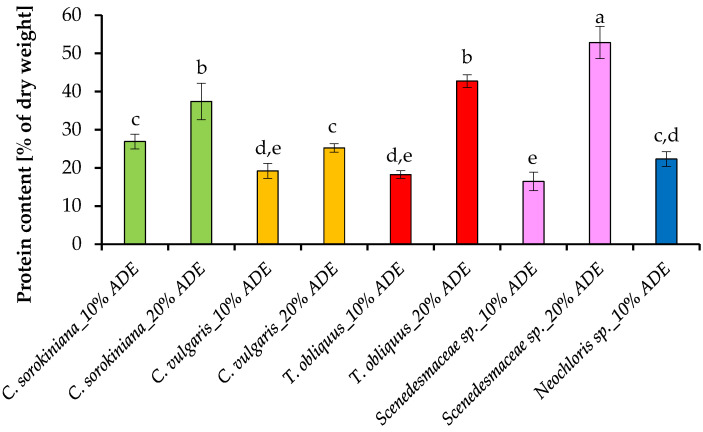
Protein content in algal cells cultured under different conditions. Means that do not share a letter are significantly different (ANOVA, Tukey method, α = 0.05).

To reveal the effect of ADE concentrations on the composition of algal cells, the content of the proteins in the tested strains was evaluated. The accumulation of proteins by microalgal cells grown in the photobioreactor at different concentrations of ADE was determined at the end of the growth, and the obtained data are summarized in Figure 7.

Among the chemical components of algal cells, proteins are attracting great attention for application in various industries. The cultivation of *C. sorokiniana* EZ-07 in a medium based on 10% ADE made it possible to obtain a biomass concentration containing up to 26.9 ± 1.9% of protein, while in the variants with 20% ADE, the protein content was significantly higher, with a maximum value of 37.4 ± 4.7% of the dry weight. Similar trends were observed for other cultures: a higher nitrogen level in ADE led to a higher protein content in the cells. Thus, the final protein values were as follows: 19.2 ± 2.0% and 25.2 ± 1.1% for *C. vulgaris* SB-M4; 18.2 ± 1.0% and 42.7 ± 1.7% for *T. obliquus* EZ-K8; 16.4 ± 2.4% and 52.8 ± 4.2% for *Scenedesmaceae* sp. EZ-B1 cultured in 10% ADE and 20% ADE-containing experiments, respectively. The average content of protein in the cells of *Neochloris* sp. EE-K3 when it was cultivated in 10% ADE reached 22.3 ± 1.9%. Our findings are consistent with those of other studies but these were for other strains and cultivation conditions [35].

In the present study, the protein content in algal cells during their growth at 10% effluent loading varied from 16% to 27% of dry weight, which indicates an insufficient level of available nitrogen in the medium, while at 20% effluent loading, the protein content was higher (up to 53% of dry weight), which indicates a satisfactory level of nitrogen in these experiments. A previous report noted that cultivation of *C. sorokiniana* strains in 10% ADE resulted in lower protein content in cells compared to results obtained during cultivation in synthetic media (24–34% versus 45–51%) [30]; however, no significant differences in protein content were observed between the control medium and ADE-based medium for another strain *C. sorokiniana* (37–42%) [22]. Other studies have shown that the biomass of *C. vulgaris* after cultivation in undiluted anaerobically treated swine wastewater [34] and treated domestic wastewater [36] contained up to 40% proteins. The result obtained in another research demonstrated that the highest protein content (up to 51%) can be obtained during cultivation of *Scenedesmus* sp. in diluted wet market wastewater [37]. Our study indicates that an increase in anaerobic digester effluent level stimulates the tested algal strains to accumulate a higher protein level in their cells, indicating the need to select the growth conditions to obtain more proteins that may be suitable for use as animal feed.

Finally, researchers in this field consider certain microalgal species, which can efficiently grow and effectively treat wastewater, agricultural waste, sludge, and residual digestate, as water treatment agents. This indicates the need for additional research to improve the wastewater treatment biotechnologies, the accumulation of biomass, and the production of important products during the cultivation of microalgae in various digestates.

## 3. Materials and Methods

### 3.1. Microalgal Strains: Isolation and Identification

Five different species of green microalgae were tested in this study, including *Chlorella vulgaris* SB-M4 (*Chlorellaceae*), *Chlorella sorokiniana* EZ-07 (*Chlorellaceae*), *Tetradesmus obliquus* EZ-K8 (*Scenedesmaceae*), *Scenedesmaceae* sp. EZ-B1 (*Scenedesmaceae*), and *Neochloris* sp. EE-K3 (*Neochloridaceae*). They were isolated from the water reservoirs of Kazan (the Republic of Tatarstan, Russia) in 2020–2021. Microalgae were identified by molecular analysis of the ribulose bisphosphate carboxylase (*rbcL*) large subunit gene. The methods of identification of microalgae were previously described by Ziganshina et al. [38]. The sequences were compared to public databases using BLAST program. The phylogenetic tree based on the *rbcL* gene sequences was created with the MEGA 7 software using the neighbor-joining method [23]. The *rbcL* gene sequences were deposited in the GenBank database under accession numbers OP909988–OP909991.

### 3.2. Pre-Culture Conditions

Cultures were maintained on solid Bold’s basal medium (BBM) [24] with ampicillin and kanamycin to reduce the risk of bacterial contamination (10 µg and 50 µg per 1 mL of medium, respectively). BBM was also used as a medium for the preparation of the inoculum. Microalgal inoculums for subsequent cultivation in a Labfors 4 Lux photobioreactor (Infors HT, Bottmingen, Switzerland) were grown in 250 mL Erlenmeyer glass flasks on a rotary shaker at 120 rpm, at a temperature of 28 °C, and 400 μmol photons m^−2^ s^−1^. The cells for each set of experiments were cultured to reach the stationary growth phase, then separated from the medium by centrifugation at 5000× *g*, washed with sterile K-Na-phosphate buffer (pH 7.0), and added into the photobioreactor with an OD_750_ (optical density at 750 nm) of 0.1. All procedures for following experiments in the photobioreactor were performed under aseptic conditions.

### 3.3. Experimental Organization: Effluent-Based Media Preparation 

Anaerobic digester effluent, as residue of biogas production from distiller grains with solubles and cow manure, was tested as an available and cheap source of nutrients for cultivation of various green microalgae instead of mineral medium. Laboratory scale experiments were conducted with two concentrations of the effluent obtained from laboratory anaerobic biogas reactors. Before cultivation of the microalgae, the initial digestate was centrifugated at 10,000× *g* for 10 min to obtain a liquid medium and then diluted with sterile deionized water to reach 10% and 20% (% *v*/*v*). This treatment was necessary to remove particles and most microorganisms and to improve light permeability. The initial and treated digestate was characterized by measuring pH, total solids, volatile solids, concentrations of total volatile fatty acids, and ammonium, as described in detail previously [11,12,39]. It was further stored at a temperature of +4 °C to ensure stability and prevent microbial development before cultivation of the microalgae. The pretreated ADE contained ammonium as a main source of nitrogen and was additionally supplemented with main ions, such as phosphate, sulfate, and potassium as previously described by us [21].

### 3.4. Cultivation in the Photobioreactor

Microalgae were grown in the 3.6 L Labfors 4 Lux photobioreactor with a working volume of 2.6 L with controlled luminous flux levels. Cultures were grown at 28 °C, with sparging of atmospheric air/carbon dioxide (98/2, *v*/*v*), under an illumination of 800 μmol photons m^−2^ s^−1^ (measured on the surface of vessel), and a 16 light: 8 dark photoperiod. Culture turbulence was provided by agitation at 120 rpm, whereas aeration (0.8 L min^−1^) was provided by a compressor. A thermal mass flow controller (Vögtlin Instruments, Aesch, Switzerland) was used to add carbon dioxide into the photobioreactor (air and CO_2_ were mixed before addition into the reactor). The pH of the media was measured with an EasyFerm Plus PHI K8 200 electrode (Hamilton, OH, USA). If foaming was detected, a sterile 2% solution of antifoam (Antifoam B, Sigma-Aldrich, St. Louis, MO, USA) was added to the reactor. The pH, agitation, temperature, CO_2_ flow, pressure inside the reactor, percentage of O_2_ and CO_2_ released, and light intensity were measured automatically using various Infors devices.

### 3.5. Growth Measurements and Analytical Methods

Microalgal growth was measured at 750 nm using a Lambda 35 spectrophotometer (Perkin Elmer, Singapore). Cell suspensions were diluted prior to measurements to receive a final OD_750_ of less than 0.4 for measurements. To determine the cell density, the samples were also observed under a light microscope using a counting chamber.

In addition, dry weight of biomass in middle of experiments was analyzed (calculated as the difference between the weight of a 10 mL tube with washed and dried microalgal pellet and the weight of the 10 mL tube without biomass). For the final weight measurement, the entire biomass was collected.

The biomass productivity (g L^−1^ day^−1^) was calculated as described previously [40]. The final dry weight of the produced biomass (g L^−1^) and the level of volatile solids (g L^−1^) were analyzed as described previously [21,22].

The composition of algal biomass was analyzed for total pigments and protein as previously described in detail [21,22].

Ammonium was measured in the culture supernatant every day by using Nessler’s reagent (Sigma-Aldrich, St. Louis, MO, USA). Phosphate and sulfate concentrations in an initial effluent were measured using a Dionex ICS-900 Ion Chromatography System (Thermo Fisher Scientific, Wilmington, DE, USA) equipped with an IonPac AG22 (4 × 50 mm) guard column and an IonPac AS22 (4 × 250 mm) analytical column as previously described [21,22,38].

All technical measurements were carried out in triplicate with two biological replicates of each experiment. The data is presented as the mean and the standard deviation.

The Tukey method and 95% confidence were performed on the experimental data to demonstrate the data’s statistical significance (Minitab software version 20.2.0).

## 4. Conclusions

This study demonstrated that anaerobic digester effluent can serve as a growing medium for different species of green microalgae, and protein production and wastewater treatment are simultaneously possible. Among the various species of green algae, members of the genus *Chlorella* have been found to be well suited for cultivation in media with elevated levels of anaerobic digester effluent. *C. sorokiniana* and *C. vulgaris* demonstrated a high degree of ammonium removal from the growth medium. In addition, an increase in the concentration of effluent stimulated the accumulation of proteins by microalgae. This approach, based on the introduction of microalgae into the treatment of by-products of biogas reactors, can be considered an environmentally friendly and economical option for the production of high-protein animal feed.

## Figures and Tables

**Table 1:** Characteristics of an initial effluent from anaerobic biogas reactors.

Parameter	Total Solids, %	Volatile Solids, %	Volatile Organic Acids, g L^−1^	NH_4_^+^, g L^−1^	PO_4_^3–^ mg L^−1^	SO_4_^2–^ mg L^−1^	pH
Value	5.5 ± 0.14	3.7 ± 0.11	0.37 ± 0.05	1.12 ± 0.07	74.1 ± 4.1	5.3 ± 1.1	7.97 ± 0.04

**Table 2:** Characteristics of tested microalgal strains when grown in ADE-based media.

Strain	Treatment	Final Dry Weight (g L^−1^)	Biomass Productivity (g L^−1^ Day^−1^)	Volatile Solids (g L^−1^)	Final Cell Concentration (×10^6^ Cells mL^−1^)	Maximum Carotenoids (mg L^−1^)
*C. sorokiniana* EZ-07	10% ADE	1.90 ± 0.14 ^c,d^	0.28 ± 0.02 ^a,b^	1.81 ± 0.08 ^c,d^	270.5 ± 18.2 ^b^	7.3 ± 0.5 ^c^
	20% ADE	2.81 ± 0.10 ^a,b^	0.30 ± 0.02 ^a,b^	2.62 ± 0.12 ^a,b^	380.1 ± 24.2 ^a^	12.0 ± 0.3 ^b^
*C. vulgaris* SB-M4	10% ADE	2.13 ± 0.13 ^b,c,d^	0.27 ± 0.02 ^b^	2.05 ± 0.10 ^b,c^	91.3 ± 10.3 ^c,d^	8.0 ± 0.9 ^c^
	20% ADE	3.26 ± 0.18 ^a^	0.26 ± 0.01 ^b^	2.92 ± 0.14 ^a^	132 ± 8.4 ^c^	18.5 ± 1.4 ^a^
*T. obliquus* EZ-K8	10% ADE	2.33 ± 0.15 ^b,c^	0.27 ± 0.02 ^b^	2.20 ± 0.12 ^b,c^	109.1 ± 9.4 ^c,d^	6.6 ± 0.3 ^c^
	20% ADE	1.07 ± 0.09 ^e^	0.08 ± 0.01 ^d^	0.99 ± 0.05 ^e^	72.1 ± 5.3 ^c,d^	7.3 ± 0.7 ^c^
*Scenedesmaceae* sp. EZ-B1	10% ADE	2.31 ± 0.12 ^b,c^	0.35 ± 0.02 ^a^	2.21 ± 0.08 ^b,c^	67.3 ± 7.3 ^c,d^	7.9 ± 1.0 ^c^
	20% ADE	1.46 ± 0.14 ^d,e^	0.15 ± 0.01 ^c^	1.37 ± 0.07 ^d,e^	50.1 ± 4.2 ^d^	8.3 ± 0.8 ^c^
*Neochloris* sp. EE-K3	10% ADE	2.17 ± 0.14 ^b,c^	0.24 ± 0.02 ^b^	2.09 ± 0.12 ^b,c^	64.5 ± 8.2 ^d^	6.8 ± 0.4 ^c^
	20% ADE	ND	ND	ND	ND	ND

Different superscripts indicate differences between the treatments (ANOVA, Tukey method, α = 0.05). Biomass productivity was calculated from the final dry weight. Means that do not share a letter are significantly different. ND—not determined.

## Data Availability

The data presented in this study are available on request from the corresponding author.
